# Oncological safety of intrafascial nerve-sparing radical prostatectomy compared with conventional process: a pooled review and meta-regression analysis based on available studies

**DOI:** 10.1186/s12894-019-0476-2

**Published:** 2019-05-27

**Authors:** Xiao Wang, Yiqi Wu, Jia Guo, Hui Chen, Xiaodong Weng, Xiuheng Liu

**Affiliations:** Department of Urology, Renmin Hospital of Wuhan University, 238Jiefang Road, Wuhan, 430060 People’s Republic of China

**Keywords:** Oncological safety, Radical prostatectomy, Intrafascial nerve-sparing, Systematic review, Positive surgical margins

## Abstract

**Background:**

Intrafascial prostatectomy was a modified technique from the conventional nerve-sparing surgery in order to improve patients’ post-surgical continence and erectile function; however, ongoing controversy exists regarding the oncological safety of this technique. In this study we aimed to provide a critical and pooled analysis based on published literatures regarding the oncological outcomes after intrafascial nerve-sparing prostatectomy.

**Methods:**

Database searches were performed for published articles till June 2018 on PubMed. Three reviewers screened fulfilled papers and extracted data independently. Main outcome was the positive surgical margins (PSMs) rates stratified by pathological stages. We performed both one-arm and comparative meta-analysis to evaluate the oncological safety of intrafascial technique. Moreover, we built meta-regression models to assess the confounding factors.

**Results:**

We retrieved a total of 117 records after electronic search, of which 21 studies were finally included in this review. There were 15 controlled studies and 6 surgical series. Our one-arm meta-analysis demonstrated that the total PSM rates after intrafascial techniques ranging from 2.2 to 35%, with a pooled rate of 14.5% on average (480 of 3151 patients, 95% confidence interval[CI]: 11.2–17.5%). Meta-regression model showed that patients’ age, pT2 cancer percentage and Selection Score of Oncological Safety (SSOS) were significantly associated with total PSM rate; moreover, each 1 point of SSOS could decrease the total PSM rate by 1.3% on average. Comparative meta-analysis demonstrated that there was no significant difference between intra- and inter-fascial group regarding PSM rates.

**Conclusions:**

With stringent case selection and when performed by experienced surgeons, intrafascial prostatectomy could offer an acceptable or, at least, equivalent PSM rate compared with the conventional interfascial approach. Preoperative SSOS more than 7 points could be considered as an indication of intrafascial radical prostatectomy.

**Electronic supplementary material:**

The online version of this article (10.1186/s12894-019-0476-2) contains supplementary material, which is available to authorized users.

## Background

Radical prostatectomy is recommended as an effective treatment for clinically localized prostate cancer [1, 2], and used to cure patients with low/intermediate risk of disease [3]. Based on the updated anatomy understandings, the approaches to preserve peri-prostatic nerves have undergone many modifications from the conventional nerve-sparing surgery [4]. Intrafascial technique was a kind of refinements, characterized by developing a dissection plane medially/internally to the prostatic fascia, in order to maximally preserve peri-prostatic nerves and to enhance the post-surgical recovery of continence and potency. This technique is now applied worldwide in combination with different surgical approaches and procedures [5, 6].

Controversy about the intrafascial nerve-sparing technique has persisted since its introduction [7]. Most doubts were concentrated at its oncological safety, considering the necessity of removing all fascial coverings of the prostatic surface in resecting a tumor. It is generally recognized that the greater extent of structures spared, the higher is the risk of residual tumor; therefore, some surgeons would be concerned that intrafascial dissection would compromise its oncological safety and incur a risk of a higher rate of positive surgical margins (PSMs) [8].

There were obvious variations regarding the oncological results reported by different surgeons, which may be influenced by factors such as surgeon characteristics, surgical procedures, patient inclusion criteria, and outcome assessment methods. The purpose of this study was to critically summarize existing clinical trials and provide a detailed and comprehensive assessment of the oncological findings of intrafascial prostatectomy to guide urologists in selecting the appropriate technique.

## Methods

### Inclusion criteria

Inclusion criteria were set according to PICOS (patients, intervention, comparison, outcomes, and study design) principle as presented in Table 1. This review included trials designed as surgery series or controlled studies. Included studies had at least one arm that is performed using intrafascial techniques, including veil technique and other techniques approaching fascial planes close to the prostatic capsule and internal to the prostatic fascia, regardless of the types of surgery including retropubic radical prostatectomy (RRP), laparoscopic radical prostatectomy (LRP) and robot-assisted laparoscopic radical prostatectomy (RALRP). Study paralleling convetional interfascial technique was included in the comparative analysis as a control study. We excluded studies from the comparative assessment, including extra-fascial or wide-dissection or non-nerve-sparing prostatectomy, and for these studies we only extracted data from the intrafascial group.

**Table 1:** PICOS principle of inclusion criteria

Criteria	Description
Patients	Adult men diagnosed as prostate cancer undergoing radical prostatectomy
Intervention	Intrafascial nerve-sparing radical prostatectomy, including Veil, Leipzig, curtain dissection, or other techniques approaching fascial planes on the surface of the prostatic capsule or medial/internal to the prostatic fascia, regardless of surgery types
Comparison	Conventional interfascial nerve-sparing prostatectomy, regardless of surgery types
Outcomes	PSM rates stratified by pathological stages
Study design	Surgical series or prospective/retrospective controlled studies, including RCTs

*PSM* positive surgical margin, *RCTs* randomized controlled trials

### Data sources and searches

Database searches were performed for published articles till June 2018 on PubMed. The following keywords were used across the “Title” and “Abstract” field including: (“intrafascial” OR “veil” OR “curtain dissection” OR “incremental nerve sparing” OR “high anterior release”) AND “radical prostatectomy”. Study characteristics were in accordance with our PICOS principle. There were no restrictions on the reporting characteristics of the publication status or language. In addition, there were no restrictions on the time of surgery or the date of publication. We manually checked the reference list of the included studies to further identify other relevant studies. Three reviewers (JG, HC, XW) independently screened the titles, abstracts and keywords of each search article. If the study met the inclusion criteria, we screened the full text for further evaluation. We excluded duplicate publications or superficially reported studies. Disagreements were resolved through open discussion.

### Data extraction and synthesis

Data were independently extracted by three reviewers (JG, HC, XW) using standard formats, including study characteristics, patient characteristics, surgical information and outcomes. The authors of the original study were consulted for the missing data if needed. As surgical margins were confounded by patient selection, we evaluated and scored the patients’ preoperative risk level according to the nomogram of Partin tables [9] along with D’Amico’s study [10]. The scoring scale shown in Table 2 is stratified into 4 parts including clinical tumor stage, preoperative PSA level, Gleason score and invaded cores percentage. The scores of the 4 parts were summed up to the Selection Score of Oncologic Safety (SSOS). A high score indicated a more rigorous patient selection criteria and low risk of extraprostatic extension and margin involvement.

**Table 2:** Scoring scale of Selection Score of Oncologic Safety

Section	Clinical T stage						Preoperative PSA level (ng/ul)					
Item	T1c	T2a	T2b	T2c	T3	NS	0–2.5	2.6–4	4.1–6	6.1–10	> 10	NS
Score	5	4	3	2	1	0	5	4	3	2	1	0
Section	Biopsy Gleason score						Invaded cores percent (%)					
Item	2–4	5–6	3 + 4	4 + 3	8–10	NS	< 34	34–50		> 50		NS
Score	5	4	3	2	1	0	3	2		1		0

This scoring scale was according to the Partin table and D’Amico’s study, stratified to 4 parts including clinical stage, preoperative PSA level, Gleason score and invaded cores percent. Scores of the 4 parts were summed to the Selection Score of Oncologic Safety (SSOS). High score means the more rigorous selection criteria for patients and low risk of extraprostatic extension and margin involvement. *PSA* prostate-specific antigen, *NS* not evaluated

With regard to a comparative analysis of the PSM rate, some studies had a selection bias between the intra- and inter-fascial groups. As surgeons often doubted the oncological safety of the intrafascial technique, they restricted the application of this technique to patients with early-stage tumors and lower risk of extraprostatic extension, but employed the interfascial procedure in high-risk disease. We considered this bias as a selection imbalance and, if surgeons made no exception in the selection of patients, we judged this as selection balance.

### Bias assessment

The Cochrane Collaborative Bias Assessment Tool was used to assess the methodological quality of the included control studies. The following items were evaluated: (1) Adequate sequence generation? (2) Allocation concealment? (3) Binding? (4) Incomplete outcome data addressed? (5) Free of selective reporting? (6) Free of other bias? Each question was rated as “low risk”, “high risk” or “unclear” and three reviewers (JG, HC and XW) independently assessed each trial. We used the funnel plot to evaluate publication bias. If there is a disagreement, judgment was made through public discussion.

### Data analysis

All data extracted from the intrafascial arms were pooled using Open Meta-analyst software, stratified by surgical type. We performed one-arm meta-analysis using random effects models, and heterogeneity in the studies was assessed using Chi-square test and the I^2^ index. Meta-regression analysis was performed with total PSM rate as a dependent variable, including patient age, preservation techniques, pT2 cancer percentage, and SSOS as covariate variables. An in-depth regression analysis was performed by including each part of the SSOS and excluding each part from the SSOS.

Comparative meta-analysis was conducted with the Cochrane Collaboration Review Manager. Heterogeneity among the studies was assessed using Chi-square test and the I^2^ index statistic. When *p* > 0.1 and I^2^ < 50%, fixed-effect models were applied for the calculation of pooled effect index and only if *p* < 0.1 and I^2^ > 50%, the random-effect models were used. A comparative analysis of the PSM rates was performed by stratifying selection balance as a subgroup.

## Results

**Fig. 1:**
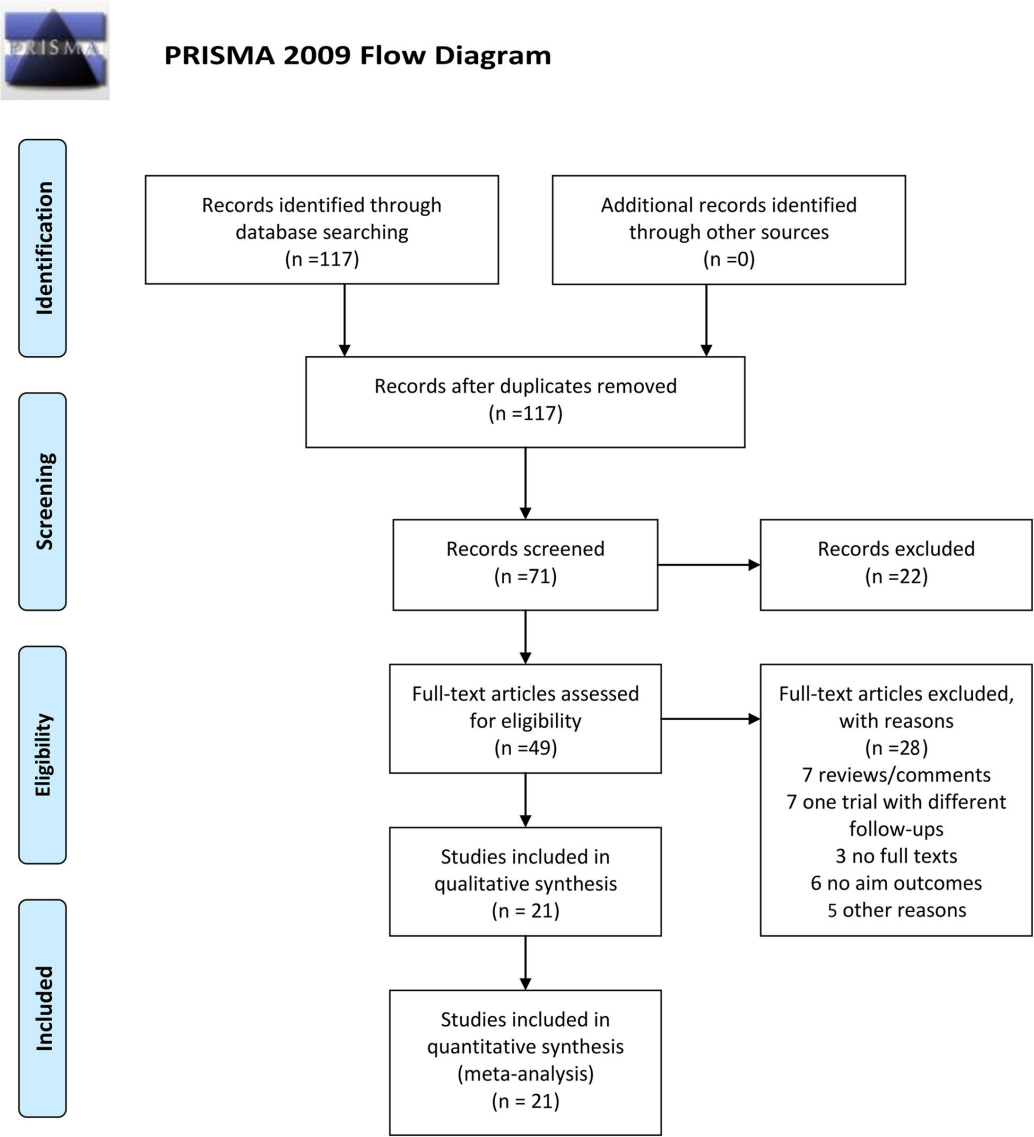
PRISMA flowchart for identification and selection of studies for this systematic review

This review was performed according to the Preferred Reporting Items of Systematic Reviews and Meta-analysis (PRISMA) statement. A total of 117 records were retrieved after electronic search strategy, which ultimately included 21 studies [6, 11–30]. Figure 1 present the PRISMA flowchart of literature searches and Table 3 provided detailed characteristics of the included studies. Among the 21 studies included, 15 were controlled studies and 6 were surgical series. Eight trials paralleling conventional interfascial technique as controlled groups were included for comparative meta-analysis. Additional file 1: Table S1 showed the risk of bias in individual studies.

**Table 3:** Characteristics of the included studies in meta-analysis

First author	Study design	Country	Surgery duration	Sample size for Intra-RP	Surgical type	Mean age in Intra-RP
Curto 2006	surgical series	France	2003.5–2005.3	425	LRP	62.0
Budaus 2009	surgical series	Germany	2005.4–2007.12	1150	RRP	63
Stolzenburg 2008	surgical series	Germany	2001.12–2007.11	150	LRP	60.2
Xylinas 2010	surgical series	France	2007.12–2008.6	50	RALRP	60.8
Asimakopoulos 2010	surgical series	Italy	2007.10–2009.3	30	RALRP	52
Khoder 2012	surgical series	Germany	2007.1–2009.12	231	RRP	63.3
Greco 2010	controlled	Germany	2005.1–2007.11	300	LRP vs RRP	61
Greco 2011	controlled	Germany	2005.1–2009.5	250	LRP	59
Asimakopoulos 2011	randomized controlled	Italy	2007.10–2008.10	128	LRP vs RALRP	60.4
Hoshi 2013	controlled	Japan	2009.1–2011.10	44	LRP	65.7
Stewart 2011	controlled	UK	2006.2–2009.12	102	LRP	61.5
Mortezavi 2012	controlled	Switzerland	2006.5–2008.8	80	RALRP	
VIP 2005	controlled	USA	2003.1–2003.12	35	RALRP	58.6
Neil 2009	controlled	UK	2000.3–2007.10	240	LRP	59
Potdevin 2009	controlled	New Jersey	2006.1–2007.12	70	RALRP	58.63
Stolzenburg 2010	randomized controlled	Germany	2004.6–2008.6	200	LRP	61
Choi 2012	controlled	Korea	2011.11–2012.4	50	LRP	66.5
Ihsan-Tasci 2015	controlled	Turkey	2009.8–2012.12	200	RALRP	60.8

*Intra-RP* intrafascial radical prostatectomy, *VIP* Vattikuti Institute Prostatectomy, *RRP* retropubic radical prostatectomy, *LRP* laparoscopic radical prostatectomy, *RALRP* robot-assisted laparoscopic radical prostatectomy

### One-arm meta-analysis

**Fig. 2:**
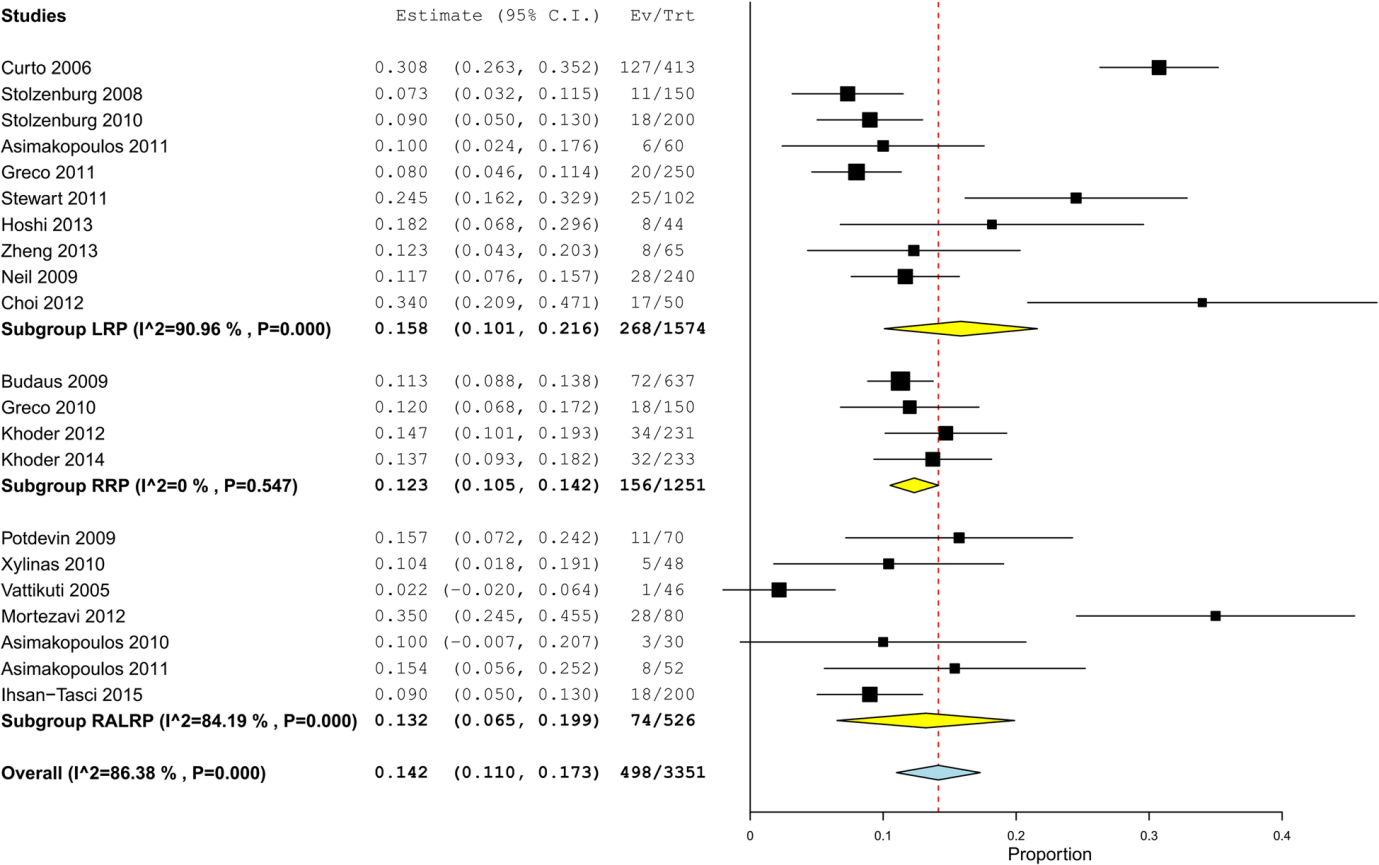
Forest plot for one-arm meta-analysis of studies adopting the intrafascial technique in terms of total PSM rate in all-stage disease stratified by surgical types. PSM, positive surgical margin; LRP, laparoscopic radical prostatectomy; RRP, retropubic radical prostatectomy; RALRP, robot-assisted laparoscopic radical prostatectomy; VIP, Vattikuti Institute Prostatectomy

Figure 2 summarized the prevalence of total PSMs recorded in the intrafascial group of the published surgical series or controlled studies. The total PSM rate ranged from 2.2 to 35%, with a pooled rate of 14.2% (498/3351, 95% confidence interval [CI]: 11.0–17.3%). It was possible to calculate the heterogeneity among the included studies as I^2^ was 86.38%. Three studies showed significantly higher PSM rates than the pooled rate, which included the studies of Curto 2006 [11], Choi 2012 [18], and Mortezavi 2012 [16], with a PSM rate of 30.8% (127/413, 95% CI: 26.3–35.2%), 34% (17/50, 95% CI: 20.9–47.1%), and 35% (28/80, 95% CI: 24.5–45.5%), respectively, whereas the VIP team reported a notably lower PSM rate of 1/46 [27]. No obvious differences could be detected among the 3 surgery types of LRP, RRP, and RALRP. PSM rates of intrafascial group stratified by pathological tumor stages could be found in Additional files 2 and 3, which showed that the pooled PSM rate was 9.7% (236/2423, 95% CI: 7.0–12.4%) in pT2 cancers (Additional file 2: Figure S1) and 44.0% (208/527, 95% CI: 34.9–53.2%) in pT3 cancer (Additional file 3: Figure S2). Overlapping higher rates could be seen in terms of the PSM rate in pT2 cancer that the studies of Curto (2006) [11] and Mortezavi (2012) [16], which also reported a significantly higher PSM rate than the pooled rate; however, this overlap was not observed with regard to the PSM rate in pT3 cancer.

### Meta-regression analysis

**Fig. 3:**
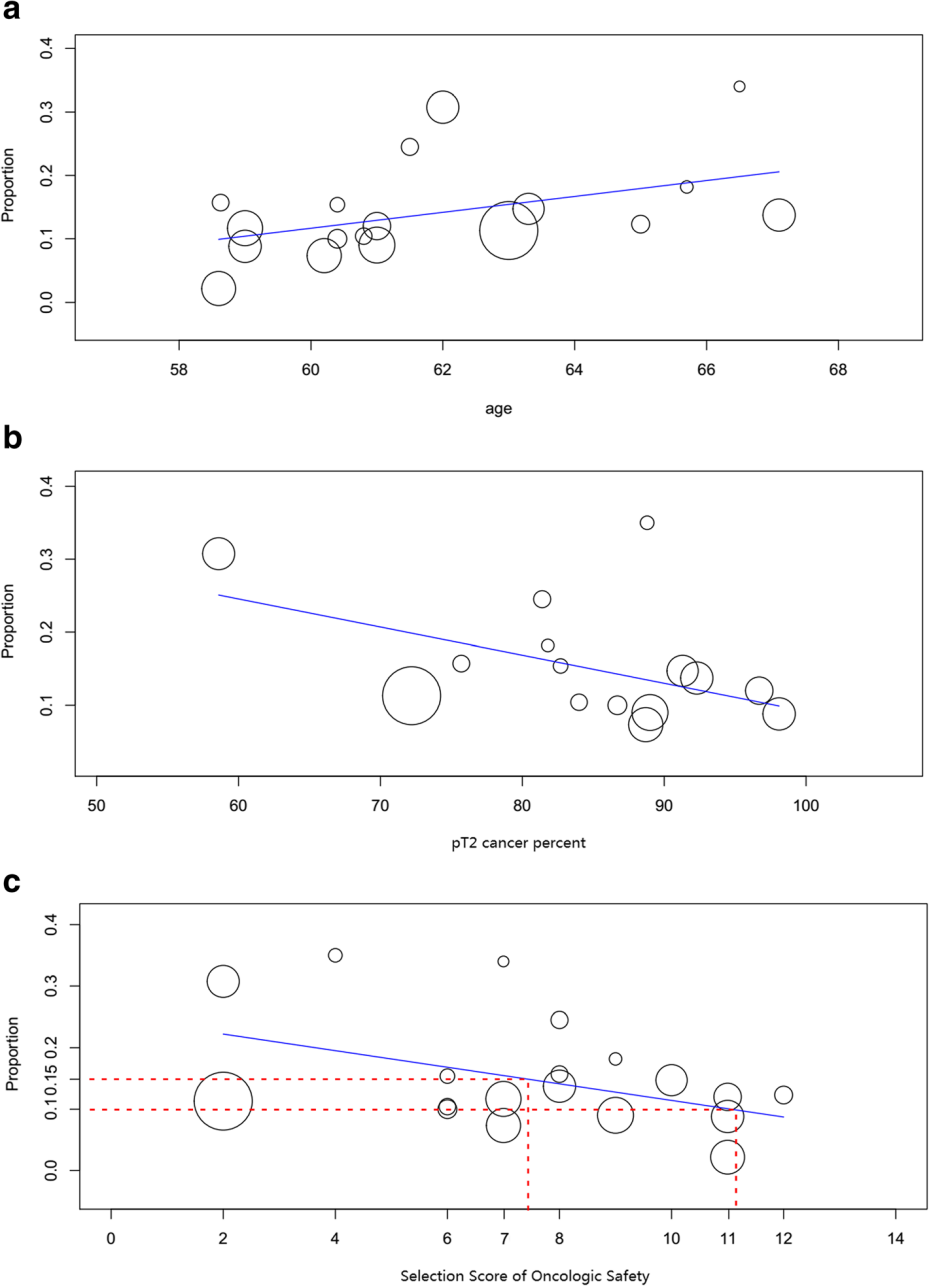
Influence of (**a**) age, (**b**) pT2 cancer percentage, and (**c**) the Selection Score of Oncologic Safety on total PSM rate in all-stage cancer following intrafascial radical prostatectomy. PSM, positive surgical margin

Table 4 summarized the result of meta-regression evaluating the impact of confounding factors affecting PSM rates. Age, pT2 cancer percentage, and SSOS were significantly associated with the total PSM rate, and age was the only factor that affected the PSM rate in pT3 cancer. Regarding the total PSM rate, the regression model demonstrated that 1 more year to the mean age could increase the total PSM rate by an average of 1.2%, while each 1 point of SSOS could decrease it by an average of 1.3%. Moreover, preservation techniques including D-fascia preservation, puboprostatic ligament sparing, selective ligation of DVC, had no significant influence on PSM rate. Figure 3 depicted the meta-regression plot to describe the effect of the confounding factors of patient age, pT2 cancer percentage, and SSOS score on total PSM rate.

**Table 4:** Meta-regression models evaluating the influence of the confounded factors to the PSM rate

Factor	Age		D-fascia preservation		Puboprostatic ligament sparing		Selective/no ligation of DVC		pT2 cancer percent		Selection Score of Oncologic Safety	
	β ± SE	*P*-value	β ± SE	P-value	β ± SE	P-value	β ± SE	P-value	β ± SE	P-value	β ± SE	P-value
Total PSM rate in all-stage	**0.013 ± 0.006**	**0.044**	−0.032 ± 0.037	0.385	−0.022 ± 0.039	0.561	0.021 ± 0.045	0.643	**−0.004 ± 0.002**	**0.012**	**−0.013 ± 0.005**	**0.012**
PSM rate in pT2 cancer	0.005 ± 0.005	0.303	0.011 ± 0.032	0.727	−0.050 ± 0.032	0.120	−0.044 ± 0.033	0.183	–	–	–	–
PSM rate in pT3 cancer	**0.041 ± 0.018**	**0.025**	−0.001 ± 0.097	0.990	0.111 ± 0.075	0.139	−0.111 ± 0.074	0.133	–	–	–	–

Each of the confounded factors including patients’ age, preservation technique(D-fascia preservation, puboprostatic ligament sparing and selective/no ligation of DVC) was included respectively in the meta-regression models to assess the influence of the confounded factors to the PSM rate. If *p*-value was less than 0.05, the cofficient and *p*-value were showed as boldface in the table. *D-fascia* Denonvilliers fascia, *DVC* dorsal venous complex, *PSM* positive surgical margin, *β* coefficient, *SE* standard error

### Comparative meta-analysis

**Fig. 4:**
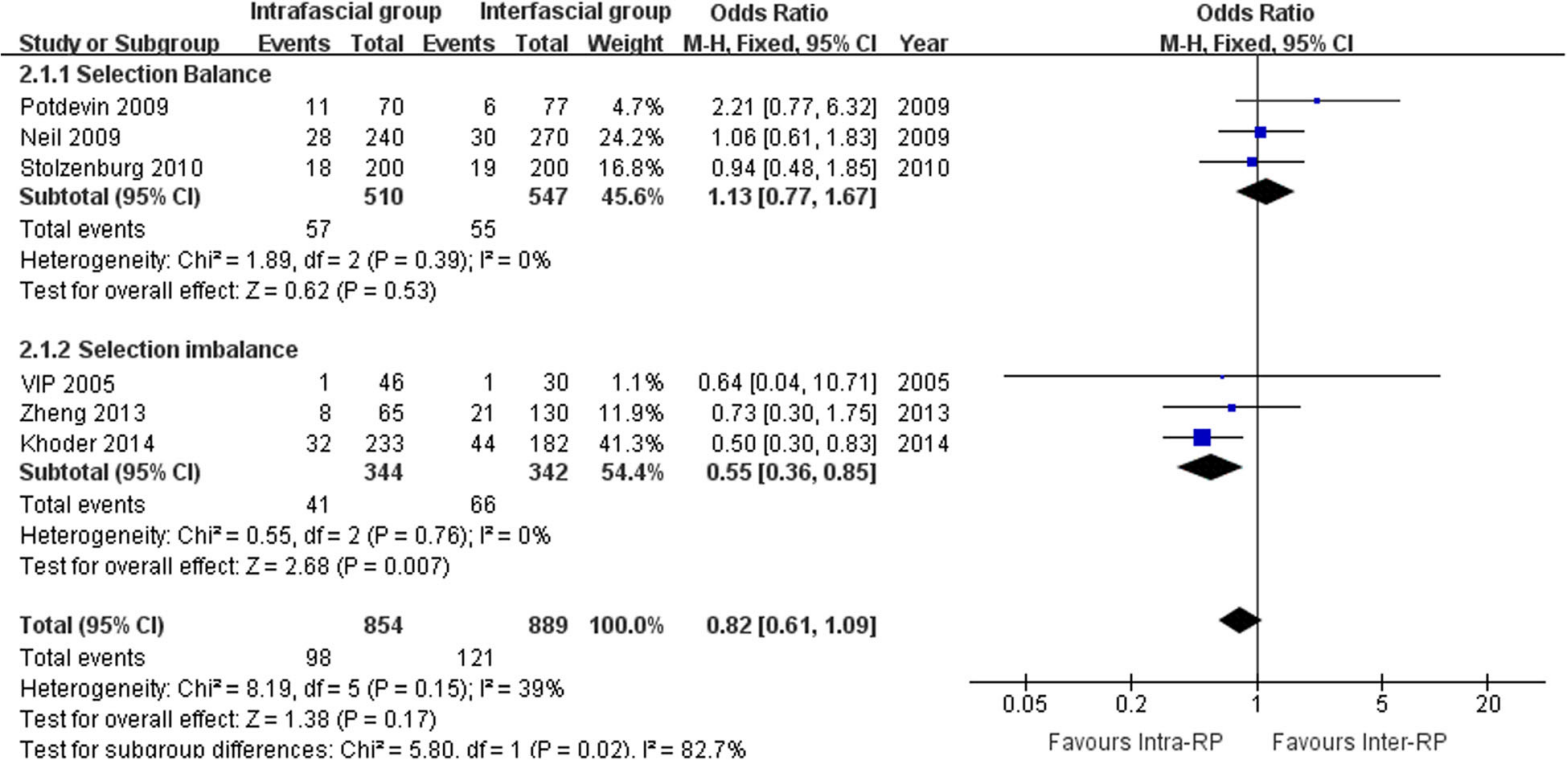
Forest plots for comparative meta-analysis of studies comparing the intrafascial with the interfascial technique in terms of the total PSM rate in all-stage cancer stratified by whether selection balance was achieved. PSM, positive surgical margin; VIP, Vattikuti Institute Prostatectomy

**Fig. 5:**
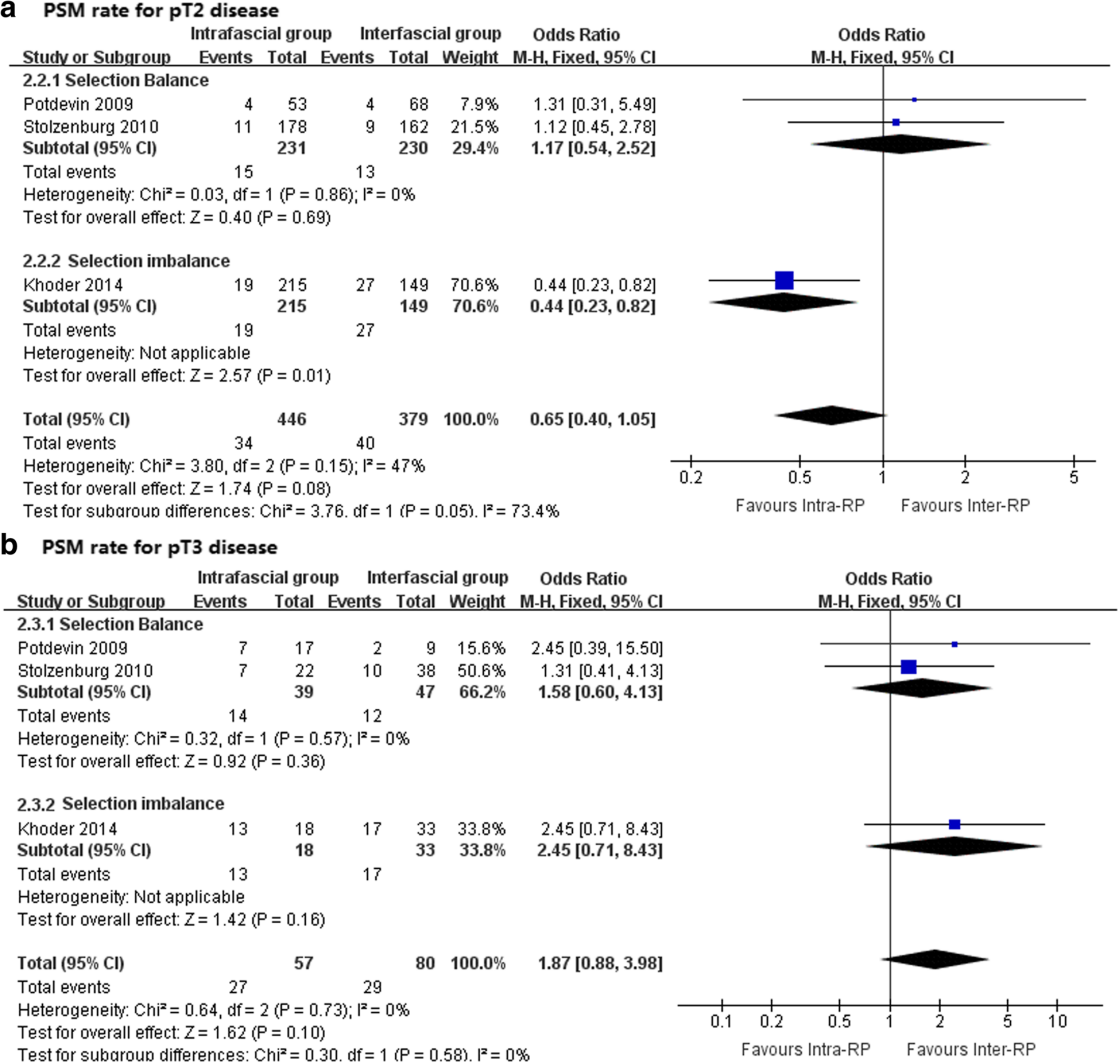
Forest plots for comparative meta-analysis of studies comparing the intrafascial with interfascial technique in terms of (**a**) PSM rate for pT2 cancer and (**b**) PSM rate for pT3 cancer stratified by whether selection balance was achieved. PSM, positive surgical margin

As shown in Figs. 4 and 5, the I^2^ standing for heterogeneity among the studies was 39, 47, and 0% in the meta-analysis of total PSM rate, PSM rate in pT2 cancer, and PSM rate in pT3 cancer, respectively, therefore, fixed-effects models were applied. None of the 3 meta-analyses demonstrated a significant difference between the intra- and interfascial groups. It is noteworthy that, in the meta-analysis of total PSM rate (Fig. 4) and PSM rate in pT2 cancer (Fig. 5a), under a balanced selection of preoperative oncologic risk, the interfascial group had a numerically lower rate compared with the intrafascial group, but interestingly, pooling the studies without a balanced baseline resulted in an observable significant difference in PSM rate in favor of the intrafascial group. This condition was not observed in the assessment of the PSM rate in pT3 (Fig. 5b), which showed consistency between the balanced and imbalanced studies, both indicating that the intrafascial group was associated with a higher PSM rate, although insignificant statistically. Table 5 provided characteristics of studies included in comparable meta-analysis reporting PSM and Additional file 4: Figure S3 showed the funnel plots for assessing the publication biases.

**Table 5:** Characteristics of studies included in comparable meta-analysis reporting PSM rate

First author	Selection balance	Criteria for Intra-RP	Criteria for Inter-RP	SSOS for Intra-RP	Percent of pT2 cancer, % (Intra-RP vs Inter-RP)	PSM rate, % (Intra-RP vs Inter-RP)		
						pT2	pT3	all-stage
VIP 2005	N	cT1c and PSA ≤ 10 and Gleason score ≤ 6	cT2a/b or PSA > 10 ≤ 20 or Gleason score = 7	11				2.2: 3.3
Zheng 2013	N	cT1/2a and PSA ≤ 10 and Gleason score ≤ 3 + 4 and positive cores≤3/12	cT2b/c or positive cores > 3/12 and PSA ≤ 10 and Gleason score ≤ 3 + 4	12				12.3: 16.2
Khoder 2014	N	cT1/2 and PSA ≤ 10 and Gleason score ≤ 6	cT1/2 and PSA ≤ 15 and Gleason score ≤ 7	8	92.3: 81.9	8.8: 18.1	72.2: 51.5	13.7: 24.2
Potdevin 2009	Y	cT1/2a and PSA ≤ 10 and Gleason score ≤ 7		8	75.7: 88.3	7.5: 5.9	41.2: 22.2	15.7: 7.8
Neil 2009	Y	cT1/2 and PSA ≤ 10 and absence of primary Gleason pattern 4/5		7				11.7: 11.1
Stolzenburg 2010	Y	cT1/2a and PSA < 10 and Gleason score ≤ 3 + 4		9	89: 81	6.2: 5.6	31.8: 26.3	9.0: 9.5

*Intra-RP* intrafascial radical prostatectomy, *Inter-RP* interfascial radical prostatectomy, *SSOS* Selection Score of Oncologic Safety, *VIP* Vattikuti Institute Prostatectomy, *Y* yes, *N* no.

## Discussion

In this study we conducted a systematic review and pooled analysis of oncological outcomes following intrafascial nerve-sparing prostatectomy. In 2012, a detailed and in-depth systematic review summarizing all surgical series of RALP between 2008 to 2011 was reported by Novara et al. [31], wherein they indicated that the prevalence of PSM after RALP was, on average, 15% in all-stage disease and 9% in pathologically localized cancer; PSM rates were similar following RARP, RRP, and LRP. The authors proposed an average PSM rate of 15 and 10% could be expected for all-stage and pT2 cancers after RALP, respectively. In this present meta-analysis, we found that the pooled PSM rate in the intrafascial group was, overall, 14.2% in all stages of prostate cancer and 9.7% in pT2 disease regardless of surgery types, which seemed to match the results of previous review. Comparative analysis revealed no significant difference in PSM rate between the intra- and inter-fascial groups, which was consistent with a previous meta-analysis [32]. From this point of view, oncological outcomes of intrafascial technique seemed acceptable, or at least not worse than with conventional approach. However, a hasty conclusion of the oncological safety of this technique should be avoided, as there are additional issues worthy of critical appraisal.

Extracapsular extension of carcinoma could lead to higher PSM rate of radical prostatectomy compared with that associated with a localized tumor. The mean percentage of pT2 cancer in the intrafascial studies included in our present review was 84.5% (range: 58.6–98.1%), which is significantly higher than that in the 17 surgical series included in Novara’s review [31] (t-test of independent samples: mean difference = − 7.91569, t = − 2.618, *p* = .014); this meant, surgeons selected more patients with localized disease to perform intrafascial approach, but only comparable total PSM rates were gained. Moreover, according to the guidelines of the European Association of Urology, radical prostatectomy could be extended for indications such as patients with intermediate-risk, localized prostate cancer with clinical stage T2b–T2c, Gleason score = 7, or PSA 10–20 ng/ml [3]. However, our review found that most surgeons applied more rigorous selection criteria against generally recommended indications for radical prostatectomy. For example, the VIP team in 2005 reported a notably low PSM rate with a robot-assisted surgical system wherein only 1 case of positive margin was detected from among 46 patients who underwent intrafascial RALRP [27]. With regard to their selection criteria, only patients with clinical T1c stage cancer combined with a Gleason score ≤ 6 and preoperative PSA ≤10 were selected for the adoption of the intrafascial technique. Therefore, the currently acceptable low PSM rate following intrafascial prostatectomy could be attributed to more stringent patient selection and, subsequently, a higher proportion of localized disease; however, the oncological safety of this technique could not be confirmed. If intrafascial dissection is carried out among patients with a high risk of extracapsular invasion, the result may be unsatisfactory. For example, in 2006, Curto et al. reported a contemporary laparoscopic surgical series wherein a surgical team experienced in over 2800 LRP performed intrafascial prostatectomies in patients with organ-confined prostate cancer (including 3 cases of cT3a disease) without other selection restrictions such as PSA level or Gleason score, for control of preoperative oncological risk. Subsequently, the percentage of pT2 cancer was only 58.6% and the overall PSM rate in all-stage tumors was as high as 30.7% (127/413) [11].

With regard to our regression model, the percentage of pT2 cancer was significantly associated with the total PSM rate, and high proportion of pT2 disease led to a low PSM rate. However, as preoperative pathological staging could not be obtained, the risk of extracapsular invasion was predicted on the basis of the patient’s preoperative information. Clinical stage, PSA level, Gleason score, and positive biopsy cores could be used as independent predictors of this risk and, therefore, we scored each factor and summed to SSOS to quantitatively evaluate this risk preoperatively. We identified a significant correlation between the SSOS and pathological pT2 percentage (Pearson Correlation = 0.749, *p* = .001). Moreover, an obvious association could be identified from our meta-regression model by including SSOS as a confounding factor, indicating that, for intrafascial prostatectomy, stringent case selection was associated with low risk of PSM and quantitatively, each 1 point of SSOS could decrease the total PSM rate by 1.3% on average. Thus, based on our regression model, the preoperative SSOS should be more than 7 points to obtain a postoperative PSM rate of 15% on average and SSOS more than 11 points meant a postoperative PSM rate of 10%. Surgeons intending to adapt this technique should take cautions when setting criteria for patient selection and we, for the first time, proposed the SSOS as an indication of intrafascial prostatectomy.

SSOS had four components, by which we used to evaluate the patients’ selection criteria for intrafascial prostatectomy of the included studies. We performed in-depth regression analysis setting total PSM rates as dependent variable by including each section and excluding each section from SSOS (see Additional file 5: Table S2). The result showed that section 1 (clinical T stage) was the most influential factor, as only section 1 could significantly affect PSM rates and when excluding section 1, the regression model became insignificant. Thus surgeons should make clinical T stage a priority when using SSOS as an indication.

A histological study by the VIP team indicated that 2/30 anterolateral zones of the prostate specimen after the Veil technique revealed capsular incision, compared with 0/40 for the standard technique [33]. In pathologically localized disease, the tumor does not invade beyond the limit of the prostatic capsule; thus, only intracapsular incision will lead to positive margins. Theoretically, intrafascial dissection should increase risk of capsular incision compared with the interfascial technique and, subsequently, with a localized tumor situated close to the capsule or having large volume, is likely to lead to PSM. This can be inferred from Curto’s surgical series [11], wherein investigators found a higher PSM rate, especially for pT2c tumors, as compared with the previous largest series; however, this difference was not present for other pathological stages.

For comparative analysis, we only included parallel-group studies including conventional interfascial nerve-sparing prostatectomy as the control. Several studies compared the outcomes of intrafascial prostatectomy with that of wide dissection or non-nerve-sparing prostatectomy, but as reported in some of these studies, wider preservation of the NVBs may be associated with a higher risk of PSM [34, 35]. Thus, for these studies, the oncological results could be confounded by the nerve-sparing technique, and, therefore, we did not include these studies in the comparative analysis. In our meta-analysis comparing intra- and inter-fascial groups, we stratified studies according to patient selection balance; however, the overall pooled result demonstrated there was no significant difference for PSM rate in all pathological stages of disease. Notably, in the study by Khoder et al. [12], patients were selected to undergo intrafascial prostatectomy only if biopsy Gleason scores were ≤ 6 and PSA ≤10 ng/mL with low tumor size; patients with relatively higher Gleason scores and PSA levels were allocated to the interfascial group, and this was considered as selection imbalance. These researchers ultimately reported that PSM rates for pT2 stage and all pathological stages were lower in the intrafascial group compared with the interfascial group; however, this advantage was not reported for pT3 cancer. From the subgroup results in our comparative analysis, a significantly lower PSM rate was identified in favor of the intrafascial technique in pT2 and all-stage disease for pooled studies with non-balanced selection criteria. All of these non-balanced studies applied additional more stringent patient selection criteria for the intrafascial technique, as compared with patient selection for the interfascial group. With regard to subgroup analysis of balanced studies, pooled results revealed a higher PSM rate, although statistically non-significant, with the intrafascial technique for pT2 and all-stage cancers. The variance between subgroups of balanced and non-balanced studies reconfirmed the crucial role of patient selection in controlling the PSM rate. In pT3 cancer, a higher PSM rate, although statistically non-significant, was detected for the interfascial group and may be attributable to insufficient sample size.

The PSM rate is known to be associated with surgeon-related characteristics, with surgical experience being the most important factor. Surgeons experienced in high-volume resections could decrease the PSM rate [36, 37]. A study evaluating the learning curve of radical prostatectomy indicated that increasing surgical experience was associated with substantial reductions in cancer recurrence: however, for LRP, the learning curve was slower than for RRP [38]. The relatively high PSM rate for LRP reported by Choi et al. in 2012 can be attributed to the learning curve [18]. In fact, the authors emphasized that, in the first 30 cases, the PSM rate was 51.7%, and then subsequently decreased to 9.5%. However, there are no published studies reporting an evaluation of PSM with the learning curve of the intrafascial technique.

Regarding on the dissection plane during procedure, surgeons supplied several technical variations, including VIP technique and Leipzig technique. We included all the intrafascial surgeries in this pooled study regardless of the variable techniques. Most authors described their dissection technique in the original manuscripts, but ignored pathological evaluation of the specimen regarding whether the utilized surgical technique was intra or interfacial nerve sparing. Thus we can only judge the classification depending on the authors’ description. As the PSM rates were affected by the dissection plane, the quality of the original trials may confound our conclusion and this is a major limitation when we included these trials in our pooled analysis.

In the present review, no obvious evidence indicative of preserving technique, including D-fascia preservation, puboprostatic ligament sparing, or selective/no ligation of DVC, was detected in conjunction with increased PSM rate. In a retrospective controlled study reported by Hoshi et al. in 2013 [15], the authors reconfirmed that the DVC preserving technique would not increase the PSM rate, as compared with the conventional intrafascial technique. Further, we could not conclusively determine any significant differences among the 3 surgical types of RRP, LRP, and RALRP from our pooled results. Overlapping results could be identified from the studies of Asimakopoulos and Greco. Asimakopoulos et al. conducted a randomized comparison between LRP and RALRP and found no statistically significant differences for the PSM rate [21]. In the study of Greco et al. comparing RRP with LRP, results demonstrated that both surgical types had similar PSM rates [24]. In terms of our regression model, age was another relevant predictor of the PSM rate, demonstrating that older patients had higher PSM rates for pT3 and all-stage cancers.

## Conclusions

In summary, intrafascial technique resulted in a total PSM rate of 14.2% for all-stage cancer and 9.7% in pT2 disease. With stringent case selection and conducted by experienced surgeons, intrafascial prostatectomy could offer an acceptable or, at least, equivalent PSM rate compared with the conventional interfascial approach. Current patient selection criteria are more stringent than those indicated for radical prostatectomy by guidelines, and we proposed SSOS as an indication of intrafascial prostatectomy. Preoperative SSOS should be more than 7 points to obtain an acceptable postoperative PSM rate of 15% on average.

## Additional files

Additional file 1: **Table S1.** Risk of bias summary of included controlled studies. Review authors’ judgments about each risk of bias item for included study. (DOCX 16 kb)

Additional file 2: **Figure S1.** Forest plots for one-arm meta-analysis of studies adopting the intrafascial technique in terms of PSM rate in pT2 disease stratified by surgical types. PSM, positive surgical margin; LRP, laparoscopic radical prostatectomy; RRP, retropubic radical prostatectomy; RALRP, robot-assisted laparoscopic radical prostatectomy. (TIF 2042 kb)

Additional file 3: **Figure S2.** Forest plots for one-arm meta-analysis of studies adopting the intrafascial technique in terms of PSM rate in pT3 disease stratified by surgical types. PSM, positive surgical margin; LRP, laparoscopic radical prostatectomy; RRP, retropubic radical prostatectomy; RALRP, robot-assisted laparoscopic radical prostatectomy. (TIF 1933 kb)

Additional file 4: **Figure S3.** Funnel plots for assessing publication biases of comparative meta-analysis of (a) total PSM rate, (b) PSM rate for pT2 disease and (c) PSM rate for pT3 disease. (TIF 5431 kb)

Additional file 5: **Table S2.** Meta-regression models evaluating each section of SSOS to the PSM rate. (DOCX 14 kb)

## Abbreviations

D-fascia: Denonvilliers fascia
DVC: Dorsal venous complex
LRP: Laparoscopic radical prostatectomy
NS: Not evaluated
OR: Odds ratio
PICOS: Patients, intervention, comparison, outcomes, and study design
PRISMA: Preferred reporting items of systematic reviews and meta-analysis
PSA: Prostate-specific antigen
PSMs: Positive surgical margins
RALRP: Robot-assisted laparoscopic radical prostatectomy
RRP: Retropubic radical prostatectomy
SSOS: Selection score of oncologic safety
VIP: Vattikuti Institute Prostatectomy
