# Supplementary material for: Oncological safety of intrafascial nerve-sparing radical prostatectomy compared with conventional process: a pooled review and meta-regression analysis based on available studies
Source: BMC Urol. 2019 May 27;19:41. doi: 10.1186/s12894-019-0476-2 (PMC6537360; doi:10.1186/s12894-019-0476-2)
Supplement: Supplementary file 1 — Table S1. Risk of bias summary of included controlled studies. Review authors’ judgments about each risk of bias item for included study. (DOCX 16 kb) [file 12894_2019_476_MOESM1_ESM.docx]

**Table S1. Risk of bias summary of included controlled studies**

| Study ID | Adequate sequence generation? | Allocation concealment? | Blinding of participants and personnel? | Blinding of outcome assessment? | Incomplete outcome data addressed? | Free of selective reporting? | Free of other bias? |
| --- | --- | --- | --- | --- | --- | --- | --- |
| VIP 2005 | H | H | U | U | L | L | L |
| Neil 2009 | H | H | U | U | U | L | L |
| Potdevin 2009 | H | H | U | U | L | L | L |
| Stolzenburg 2010 | L | U | U | U | L | L | L |
| Choi 2012 | H | H | U | U | U | L | L |
| Ko 2013 | H | H | U | L | U | L | L |
| Zheng 2013 | H | H | U | U | H | L | L |
| Khoder 2014 | H | H | U | U | U | L | L |

Review authors' judgments about each risk of bias item for included study. L = low risk; H = high risk; U = unclear.
