# Supplementary figures and images for: Oncological safety of intrafascial nerve-sparing radical prostatectomy compared with conventional process: a pooled review and meta-regression analysis based on available studies
Source: BMC Urol. 2019 May 27;19:41. doi: 10.1186/s12894-019-0476-2 (PMC6537360; doi:10.1186/s12894-019-0476-2)

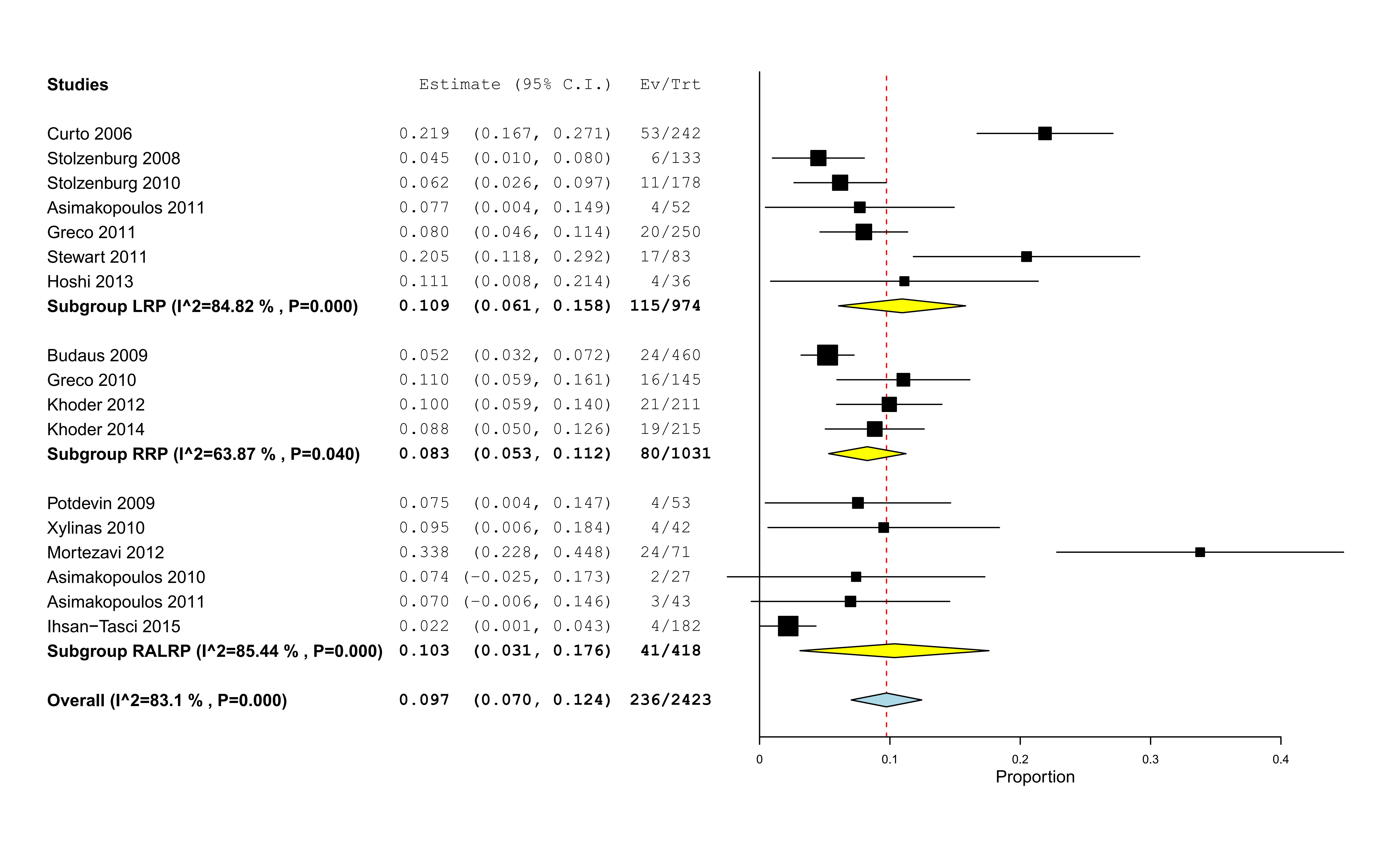

Supplement: Supplementary file 2 — Figure S1. Forest plots for one-arm meta-analysis of studies adopting the intrafascial technique in terms of PSM rate in pT2 disease stratified by surgical types. PSM, positive surgical margin; LRP, laparoscopic radical prostatectomy; RRP, retropubic radical prostatectomy; RALRP, robot-assisted laparoscopic radical prostatectomy. (TIF 2042 kb) [file 12894_2019_476_MOESM2_ESM.tif]

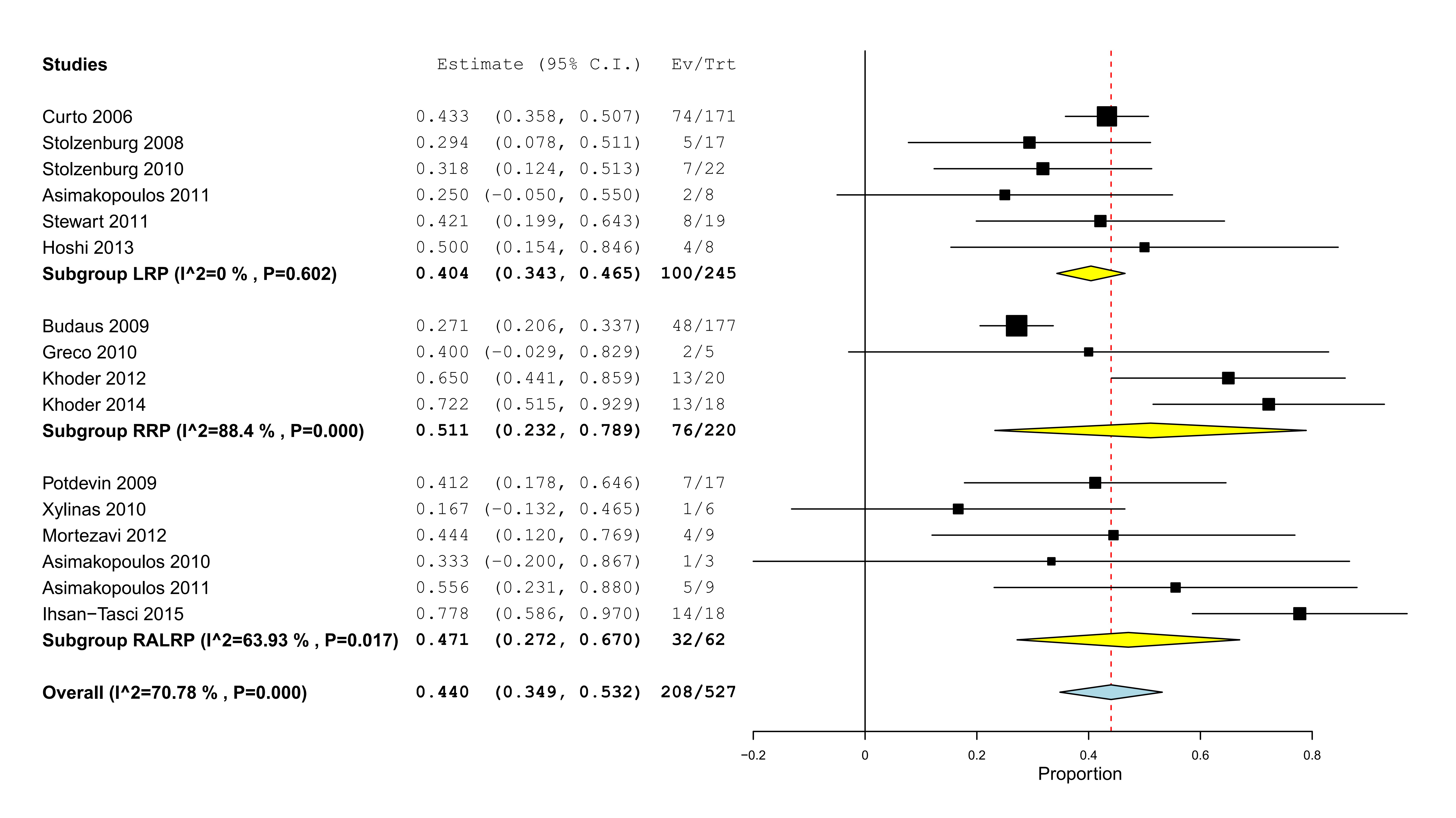

Supplement: Supplementary file 3 — Figure S2. Forest plots for one-arm meta-analysis of studies adopting the intrafascial technique in terms of PSM rate in pT3 disease stratified by surgical types. PSM, positive surgical margin; LRP, laparoscopic radical prostatectomy; RRP, retropubic radical prostatectomy; RALRP, robot-assisted laparoscopic radical prostatectomy. (TIF 1933 kb) [file 12894_2019_476_MOESM3_ESM.tif]

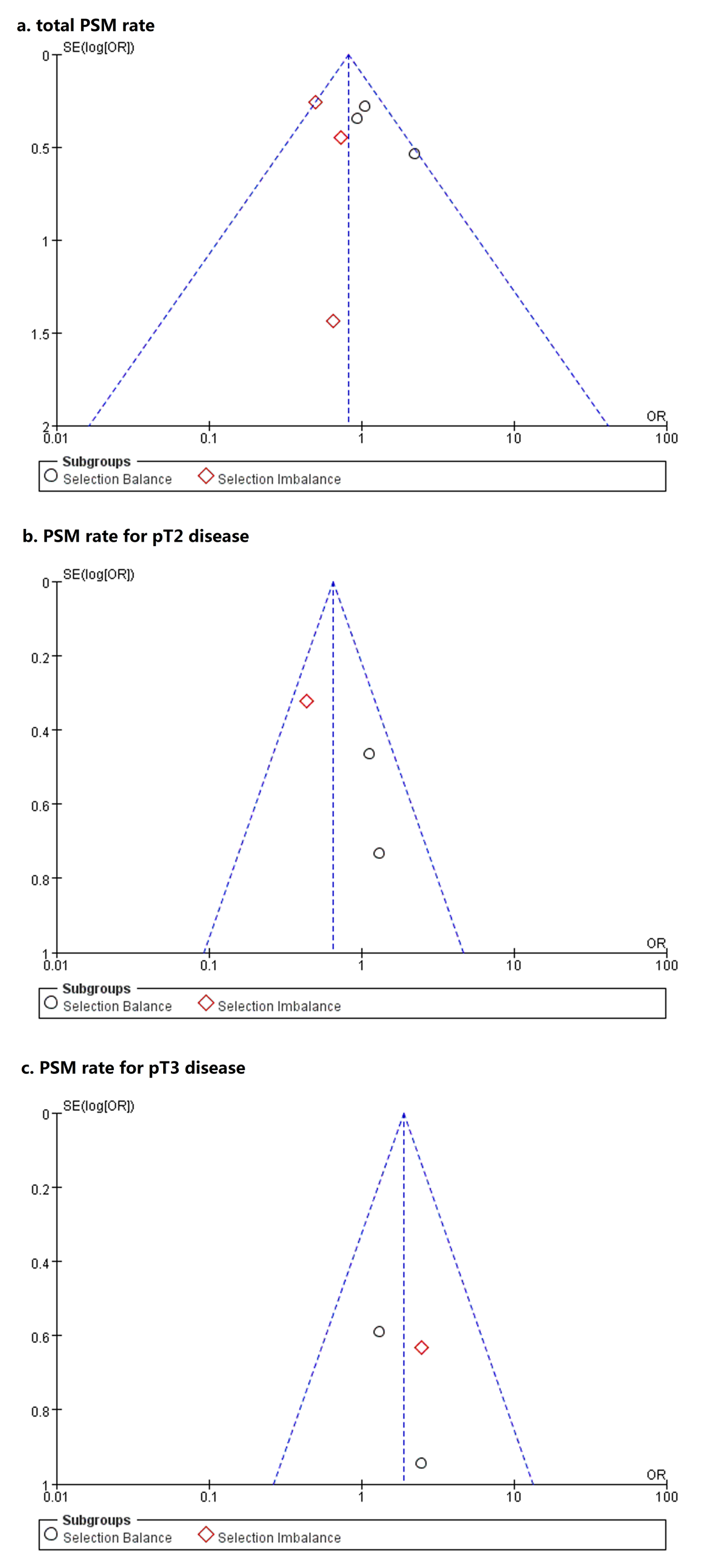

Supplement: Supplementary file 4 — Figure S3. Funnel plots for assessing publication biases of comparative meta-analysis of (a) total PSM rate, (b) PSM rate for pT2 disease and (c) PSM rate for pT3 disease. (TIF 5431 kb) [file 12894_2019_476_MOESM4_ESM.tif]
