# Supplementary material for: Oncological safety of intrafascial nerve-sparing radical prostatectomy compared with conventional process: a pooled review and meta-regression analysis based on available studies
Source: BMC Urol. 2019 May 27;19:41. doi: 10.1186/s12894-019-0476-2 (PMC6537360; doi:10.1186/s12894-019-0476-2)
Supplement: Supplementary file 5 — Table S2. Meta-regression models evaluating each section of SSOS to the PSM rate. (DOCX 14 kb) [file 12894_2019_476_MOESM5_ESM.docx]

**Table S2. Meta-regression models evaluating each section of SSOS to the PSM rate**

| Covariate | β | Lower bound | Upper bound | SE | P-value |
| --- | --- | --- | --- | --- | --- |
| Section 1 | **-0.029** | **-0.051** | **-0.006** | **0.012** | **0.013** |
| Section 2 | -0.081 | -0.169 | 0.008 | 0.045 | 0.073 |
| Section 3 | -0.006 | -0.046 | 0.033 | 0.020 | 0.753 |
| Section 4 | -0.078 | -0.155 | 0.000 | 0.040 | 0.051 |
| SSOS excluding Section 1 | -0.011 | -0.025 | 0.004 | 0.007 | 0.152 |
| SSOS excluding Section 2 | **-0.011** | **-0.022** | **-0.001** | **0.006** | **0.039** |
| SSOS excluding Section 3 | **-0.018** | **-0.033** | **-0.003** | **0.008** | **0.021** |
| SSOS excluding Section 4 | **-0.036** | **-0.055** | **-0.018** | **0.009** | **< 0.001** |
| Total SSOS | **-0.013** | **-0.024** | **-0.003** | **0.005** | **0.012** |

Each section of SOSS was included respectively in the meta-regression models to assess the influence to the PSM rate. Moreover, SSOS was also tested by meta-regression by excluding each section respectively. PSM = positive surgical margin; β = coefficient; SE = standard error.
